# Efficacy and safety of transcranial direct current stimulation in alcohol use disorder: A randomized controlled triple‐blind trial

**DOI:** 10.1111/add.70461

**Published:** 2026-05-06

**Authors:** Benoit Trojak, Anne Sauvaget, Wissam El Hage, Thomas Wallenhorst, Benjamin Rolland, Philippe Nubukpo, Ghina Harika‐Germaneau, David Szekely, Julie Giustiniani, Marc Auriacombe, Georges Brousse, Sébastien Guillaume, Maxime Bubrovszky, Benjamin Petit, Clémence Cabelguen, Hussein El Ayoubi, Suzanne Rankin, Agnès Soudry‐Faure, Karine Goueslard, Anastasia Demina

**Affiliations:** ^1^ Service Hospitalo‐Universitaire d'Addictologie Université Bourgogne Europe, CHU Dijon Bourgogne Dijon France; ^2^ INSERM U1093, UFR STAPS Université Bourgogne Europe Dijon France; ^3^ Mouvement, Interactions, Performance, MIP, UR 4334 Nantes Université, CHU Nantes Nantes France; ^4^ CHRU de Tours Clinique Psychiatrique Universitaire Tours France; ^5^ UMR 1253, iBrain Université de Tours, INSERM Tours France; ^6^ Service de Psychiatrie Adultes et Addictologie CH Semur‐en‐Auxois Semur‐en‐Auxois France; ^7^ Service Universitaire d'Addictologie de Lyon (SUAL), Hospices Civils de Lyon, CH Le Vinatier, Lyon France unité PSR2, CRNL, UMR 5292 CNRS/1028 INSERM ‐ Université Lyon 1 Bron France; ^8^ Inserm U1094 IRD UMR270, Université de Limoges, CHU Limoges, EpiMaCT Limoges France; ^9^ Pôle universitaire de de psychiatrie adultes et addictologie (PUP3A), Centre Hospitalier Esquirol Limoges France; ^10^ CeRCA CNRS, Université de Poitiers, Université de Tours Poitiers France; ^11^ Unité de Recherche Clinique Pierre Deniker du Centre Hospitalier Henri Laborit Poitiers France; ^12^ Service de Psychiatrie Centre Hospitalier Princesse Grace Monaco; ^13^ UMR INSERM 1322 LINC Université de Franche Comté Besançon France; ^14^ SANPSY, CNRS UMR 6033 Université de Bordeaux Bordeaux France; ^15^ Pôle Inter‐Établissement d'Addictologie CHU Bordeaux et CH Charles Perrens Bordeaux France; ^16^ Service de Psychiatrie Adulte et d'Addictologie CHU Clermont‐Ferrand CNRS, Université Clermont‐Auvergne, Institut Pascal Clermont‐Ferrand France; ^17^ Service Urgence et Post‐Urgence Psychiatrique, Hôpital Lapeyronie CHRU Montpellier France; ^18^ Fédération Régionale de Recherche en Santé Mentale et Psychiatrie Hauts‐de‐France France; ^19^ Etablissement Public de Santé Mentale de l'Agglomération Lilloise St André‐lez‐Lille France; ^20^ Addiction Medicine and Psychiatry Department Nantes Université, CHU Nantes Nantes France; ^21^ Direction de la Recherche Clinique et de l'Innovation, Unité de Soutien Méthodologique à la Recherche CHU Dijon Bourgogne Dijon France

**Keywords:** alcohol use disorder, efficacy, heavy drinking days, randomized controlled trial, safety, total alcohol consumption, transcranial direct current stimulation

## Abstract

**Background and aims:**

Current treatment options for alcohol use disorder are limited. Transcranial direct current stimulation has been proposed as a therapeutic approach, but evidence remains scarce. This study aimed to compare active vs. sham transcranial direct current stimulation to evaluate its efficacy and safety in reducing alcohol consumption in a large sample of individuals with alcohol use disorder.

**Design:**

REDSTIM is a triple‐blind, randomized, sham‐controlled trial that was conducted from October 2015 to January 2022. Participants were followed up every 4 weeks for 24 weeks.

**Setting:**

Fourteen sites in France and Monaco.

**Participants:**

356 adult outpatients with alcohol use disorder were assessed for eligibility, and 337 were enrolled and randomly assigned (1:1) to receive active or sham stimulation. At baseline, the randomized participants were primarily male (60.5%) with an average age of 51.3 ± 11.3 years.

**Intervention and comparator:**

Two daily stimulation sessions (anode F4, cathode F3, 2 mA) delivered over five consecutive days vs. sham stimulation. Direct currents were applied via a pair of 0.9% NaCl‐soaked surface sponge electrodes (25 cm^2^). In the sham stimulation group, the initial ramp‐up time of 15 s (also up to 2 mA) was immediately followed by a ramp down phase of 30 seconds.

**Measurements:**

The co‐primary outcomes were the change in the number of heavy drinking days (HDD) and total alcohol consumption (TAC) over the follow‐up period. Exploratory secondary outcomes included alcohol craving, clinical and biological improvements, quality‐of‐life, mood, cognitive and safety assessments.

**Findings:**

Over 24 weeks of follow‐up, vs. sham, the active stimulation group reported statistically significant reductions in the number of HDD [−2.45 HDD/4 weeks, 97.5% confidence interval (CI) = −4.86 to −0.05, *P* = 0.022]. The reduction in TAC was not statistically significant (−5.96 g/day, 97.5% CI = −15.18 to 3.26, *P* = 0.147). The interpretation of these findings should take into account the proportion of missing data related to alcohol diary completeness and losses to follow‐up.

For secondary outcomes at 24 weeks, vs. sham, craving assessments were lower in the stimulation group (−0.36 95% CI = −0.65 to −0.07, *P* = 0.016), as were carbohydrate deficient transferrin levels (−0.33 95% CI = −0.65 to −0.01, *P* = 0.045). In the active vs. sham stimulation group, 69 (41.1%) and 62 participants (36.7%) experienced one or more adverse effects, resulting in 6 dropouts.

**Conclusions:**

Among adult outpatients with alcohol use disorder, active transcranial direct current stimulation resulted in a modest but sustained reduction in heavy drinking days over 24 weeks, while no statistically significant effect was observed for total alcohol consumption. The intervention was well tolerated.

## INTRODUCTION

Alcohol consumption is associated with considerable health losses, and alcohol use is a causal factor in more than 200 disease and injury conditions. Every year, alcohol use results in a staggering 3 million avoidable deaths. Globally, an estimated 237 million men and 46 million women suffer from alcohol use disorder (AUD), particularly in Europe (prevalence of 14.8% for men and 3.5% for women) and the Americas (11.5% and 5.1%, respectively) [1].

At the same time, AUD remains dramatically undertreated. In high‐income countries, fewer than 10% of individuals with AUD receive appropriate pharmacological and psychological care [2, 3]. In addition to fearing the stigma that can accompany treatment, the effectiveness of current treatment options is a matter of debate. The few medications approved by American and European agencies (acamprosate, naltrexone, nalmefene and disulfiram), whether for their anti‐craving properties or by causing an aversion to alcohol, are only modestly effective [3, 4, 5]. Moreover, these treatments often aim for abstinence from the outset, a goal that may not be acceptable for many patients. For these patients, a drinking reduction approach seems more acceptable and sometimes more realistic [6]. However, we need new therapeutic options for AUD using these drinking reduction protocols.

In this context, newer non‐invasive brain stimulation approaches could offer an innovative treatment option for individuals with AUD [7]. These techniques modulate the neuronal excitability of superficial brain regions and deeper structures through brain connectivity [8]. In particular, transcranial direct current stimulation (tDCS) is based on the delivery of low‐intensity electric currents through electrodes on the scalp [9]. Because it can target regions of the brain such as the dorsolateral prefrontal cortex (DLPFC), tDCS is able to modulate cerebral circuits involved in AUD. Targeting the DLPFC is supported by its established role in top‐down inhibitory control, craving regulation and reward processing [10]. In AUD, the most frequently studied montage applies anodal stimulation to the right DLPFC (F4) and cathodal stimulation to the left (F3) [11]. Translational and neuroimaging studies have shown that this montage can modulate prefrontal–limbic circuitry, including functional changes in the ventromedial prefrontal cortex [12].

Three recent meta‐analyses (2021–2024) published in the field of tDCS in AUD did not find a statistically significant effect for tDCS, possibly because of the small size of the included studies and the heterogeneous treatment protocols, which were often limited to a single stimulation session [13, 14, 15]. In addition, objective clinical outcomes like alcohol reduction were generally overlooked in favor of craving or other symptomatic outcomes. In view of the low level of evidence, large, randomized trials with longer‐term follow‐up are needed. We, therefore, performed a randomized controlled trial in a large sample over a 24‐week follow‐up period to assess the clinical benefits of tDCS on the reduction of alcohol consumption in patients with AUD.

## METHODS

### Trial design and oversight

REDSTIM is a multi‐center, triple‐blind, sham‐controlled, parallel‐group, randomized clinical trial comparing five consecutive twice‐daily sessions of active tDCS vs. sham tDCS. It was carried out in 14 public hospitals in France and Monaco from October 2015 to January 2022. Each participant received a complete description of the study and provided written informed consent. The study design has previously been published [7]. No major changes were made to the methods after trial commencement.

### Patients

Eligible participants were individuals over 18 years of age who met criteria for mild to severe AUD based on the Diagnostic and Statistical Manual of Mental Disorders Fifth edition (DSM‐5) [16]. They were seeking to reduce their alcohol consumption and had at least one previous supervised drinking reduction or abstinence attempt. This last criterion aimed to ensure that participants had attempted the usual methods to treat AUD before consenting to participate in an experimental study. Participants' sex was self‐reported, and individuals of both sexes were eligible. A complete list of inclusion and exclusion criteria is provided in Table S1 in the Supporting Information.

### Randomization and masking

Participants were randomized in a 1:1 ratio to receive the active or sham intervention. Participants, investigators and operators were blinded to treatment assignment and remained masked until all patients had completed follow‐up. The allocation was based on a minimization technique stratified by center and by patient sex. To start the session, the operator entered the attributed number into the device, but they did not have access to protocol data to preserve triple‐blinding.

### Intervention

A Starstim wireless tDCS neurostimulator (Neuroelectrics) was used for active and sham stimulation in each center. Participants were instructed to remain at rest during the stimulation sessions, without engaging in any task and without closing their eyes. The anode was placed over F4 (right DLPFC) and the cathode over F3 (left DLPFC). Participants received two consecutive 13‐minutes anodal tDCS sessions (2 mA) per day on five consecutive days. The two sessions were separated by an interval of 20 minutes. No missed sessions were rescheduled. The stimulation pattern used for both sham and active stimulation (anode F4, cathode F3; 13:20:13 minutes) was in accordance with Klauss *et al*. [17], who found that these stimulation parameters had a long‐lasting, beneficial, modulatory effect on alcohol‐use relapse at 6 months. The Starstim wireless tDCS neurostimulator includes a study mode for blinded studies that encodes sham and active stimulation. After the first week of intervention, follow‐up was scheduled every 4 weeks up to week 24.

Direct currents were applied via a pair of 0.9% NaCl‐soaked surface sponge electrodes (25 cm^2^). In the active stimulation group, each of the two sessions had a ramp‐up and ramp‐down time of 15 seconds each. With sham stimulation, the initial ramp‐up time of 15 seconds (also up to 2 mA) was immediately followed by a ramp down phase of 30 seconds. A second ramp‐up (30 seconds) and ramp‐down (15 seconds) phase were delivered 13 minutes later to signal the end of a session. Using the same time pattern as for the active stimulation group, we hoped to provide convincing sham conditions for both participants and assessors. Following the last session, patients were asked to guess whether they had received the active stimulation or not to evaluate the efficacy of the blinding. The results were sealed and were only revealed after the database was frozen.

### Trial outcomes

#### Co‐primary outcomes

In 2010, the European Medicines Agency (EMA) indicated that two primary efficacy outcomes were appropriate for alcohol reduction strategies in AUD [18]. Accordingly, two co‐primary endpoints were pre‐specified in line with this guidance: the number of heavy drinking days (HDD) over 4 weeks [more than 60 g of pure alcohol per day in men (M) and 40 g in women (W)] and total alcohol consumption (TAC) (mean daily alcohol consumption over 4 weeks, g/day) during the 24‐week follow‐up period.

Baseline HDD and TAC were estimated using the alcohol timeline follow back (TLFB) based on alcohol consumption during the 4 weeks preceding randomization [19]. During the follow‐up, participants recorded their daily alcohol consumption in a paper diary. Both co‐primary endpoints were assessed at the end of the intervention and subsequently every 4 weeks throughout follow‐up, consistent with standard AUD clinical trial procedures.

#### Secondary outcomes

All secondary outcomes were pre‐specified and are reported with their CIs. Analyses of secondary outcomes were considered exploratory and are presented as supportive evidence. Secondary outcomes included:

- Change from baseline for each 4‐week period after the intervention up to week 24 in HDD and TAC; proportion of subjects with a two‐level reduction in the World Health Organization (WHO) risk drinking level (RDL): low risk (M ≤40 g/d; W ≤20 g/d), medium risk (M ≤60 g/d; W ≤40 g/d), high risk (M ≤100 g/d; W ≤60 g/d) and very high risk (H >100 g/d; F >60 g/d); proportion of subjects with a 50%, 70% and 90% reduction in alcohol consumption and the proportion of patients who potentially achieved abstinence; level of alcohol dependence severity using alcohol dependence scale (ADS); craving/urge to drink assessments [visual analog scale (VAS) for current craving, VAS for maximum craving for past 28 days and Obsessive Compulsive Drinking Scale (OCDS)]; clinical global impression‐severity and improvement; scores for the 17‐item Hamilton Depression Rating Scale (HDRS); and quality of life (12‐item Short Form Health Survey).
- Change from baseline at week 4, week 12 and week 24 in biochemical alcohol consumption markers using γ‐glutamyl transferase, mean corpuscular volume, aspartate aminotransferase, alanine aminotransferase and carbohydrate deficient transferrin (CDT); and cognitive assessment using Montreal Cognitive Assessment (MoCA).

Adverse events were collected at each study visit. No pre‐specified list of expected events was applied. For each event, information was recorded on its type (adverse event, serious adverse event or adverse reaction), severity, causality and relationship to the study treatment or procedure, onset and resolution dates (or ongoing status), any treatments administered and any resulting study discontinuation.

### Sample size

A predictive microsimulation model showed that even an approximate 10 g/day reduction in TAC results in 1000 fewer alcohol‐related events per 100 000 individuals [20]. In this context, a sample size calculation based on an expected difference of 10 g/day in TAC between the treatment groups, with a SD of 30 g/day and with a conservative autocorrelation of 0.7 between observations in the same subject, indicates that 274 patients would be required. Considering a significance level of 2.5% (co‐primary outcome) and a power of 80%, we planned to include 340 patients (170 per group) to control for the risk of attrition or non‐initiation of the treatment. The same sample size was obtained based on an expected difference of two HDDs per month between the treatment groups.

### Trial procedures

A media campaign (local newspapers, radio and social media) was used to facilitate recruitment. During this campaign, the main inclusion and exclusion criteria were specified to increase the probability that respondents would be eligible.

The study had three phases:

1. Screening: subjects were screened for eligibility. For eligible participants, inclusion and the intervention (active or sham tDCS) were scheduled in the following weeks.
2. Inclusion and intervention: this second phase lasted 1 week. It involved the first visit during which: (1) participants were included after their eligibility was confirmed by an additional clinical examination; (2) they signed the informed consent and the ‘safety agreement’ after it was ascertained that they had a zero blood alcohol level using a breath alcohol concentration test; (3) they underwent a clinical (including alcohol TLFB) and biological baseline assessment; (4) they were randomized to active or sham tDCS in a 1:1 ratio. Next, the intervention was delivered. The first stimulation session was systematically on a Monday followed by daily sessions up to Friday.
3. Follow‐up: the third phase was a 24‐week follow‐up phase starting after the completion of the intervention. There was no treatment of any kind during this phase. A clinical visit was scheduled every 4 weeks, and the prospective daily alcohol consumption diary was collected and a clinical exam was performed at each of these visits. The visits scheduled at 4, 12 and 24 weeks after the end of the intervention included sampling to measure the biochemical markers of alcohol consumption.

### Statistical analysis

The primary analysis was conducted according to the intention‐to‐treat principle and included all 337 randomized participants. To account for intra‐individual correlation across repeated measurements, mixed models for repeated measures (MMRM) were used to evaluate the effect of the intervention on reductions in HDD and TAC from baseline to the 24‐week follow‐up. The models included site, sex, treatment group, time (categorical) and the treatment‐by‐time interaction as fixed effects, with subjects included as random effects and the baseline value of the endpoint as a covariate. Baseline HDD or TAC is a controlled (i.e. non‐random) quantitative explanatory variable because mild to severe AUD was an inclusion criterion. For secondary endpoints in the intention‐to‐treat population, similar MMRM models were used for continuous outcomes, whereas binary outcomes were analyzed using generalized linear mixed models (GLMMs) with a binomial distribution.

Both co‐primary end points were assessed after completion of the intervention sessions and then every 4 weeks during the 24‐week follow‐up period, consistent with standard follow‐up procedures in AUD trials. When alcohol diary data were incomplete within a given 4‐week period, the entire period was considered missing if fewer than 28 daily entries were available.

Primary analyses were conducted under the missing‐at‐random (MAR) assumption. Missing data were handled using MMRM, which provides unbiased estimates under the MAR assumption when all available observations are included [21]. Several sensitivity analyses were conducted to assess the robustness of the findings. First, a modified intention‐to‐treat analysis (*n* = 327) was performed, excluding 10 participants with missing data on intervention completion. Second, multiple imputation by fully conditional specification was used to account for missing alcohol diary data under the MAR assumption [22]. Fifty imputed datasets were generated to estimate the effect of treatment on TAC and HDD. Third, an adjusted linear regression analysis of the change in HDD from baseline to week 24 was conducted to assess the association between treatment allocation and change in HDD. Finally, a Δ‐adjusted pattern‐mixture model was used to evaluate the robustness of the results under a missing‐not‐at‐random (MNAR) assumption.

*Post hoc* analyses included the estimation of standardized effect sizes (Cohen's *d*) and subgroup analyses according to sex, baseline severity (DSM‐defined levels) and year of inclusion. Given the limited sample sizes within these subgroups, these analyses were considered exploratory and are reported in the Supporting Information.

Blinding integrity was assessed using the Bang blinding index, which evaluates whether participants' guesses regarding treatment allocation differ from what would be expected by chance [23].

All statistical tests were two‐sided. Although one‐sided testing had initially been planned, two‐sided tests were ultimately used because the potential effects of the intervention could plausibly be bidirectional. Consequently, the α level applied in the analyses differed from that originally specified in the statistical analysis plan.

Results of the primary analyses were presented as β coefficients with their 97.5% CI. As two primary end points (HDD and TAC) were analysed, multiplicity was addressed using a Bonferroni correction and the probability of a type I error was, therefore, set at α = 0.025 (α/2). For secondary outcomes, 95% CI were reported. All analyses were performed using SAS version 9.4 (SAS Institute).

### Ethics committee approval

Ethics committee approval was provided for France by Committee for the Protection of Persons EST III on 22 June 2015 under number 2015‐A00576–43 and by the French National Agency for the Safety of Medical Products and Devices (Agence Nationale de Sécurité des Médicaments et des Produits de Santé), and for Monaco by the Biomedical Research Ethics Advisory Committee on 16 February 2016. The study was declared to the State Minister of Monaco on 29 February 2016. The participants did not receive any financial compensation for their involvement in this trial.

## RESULTS

### Patients

**FIGURE 1:**
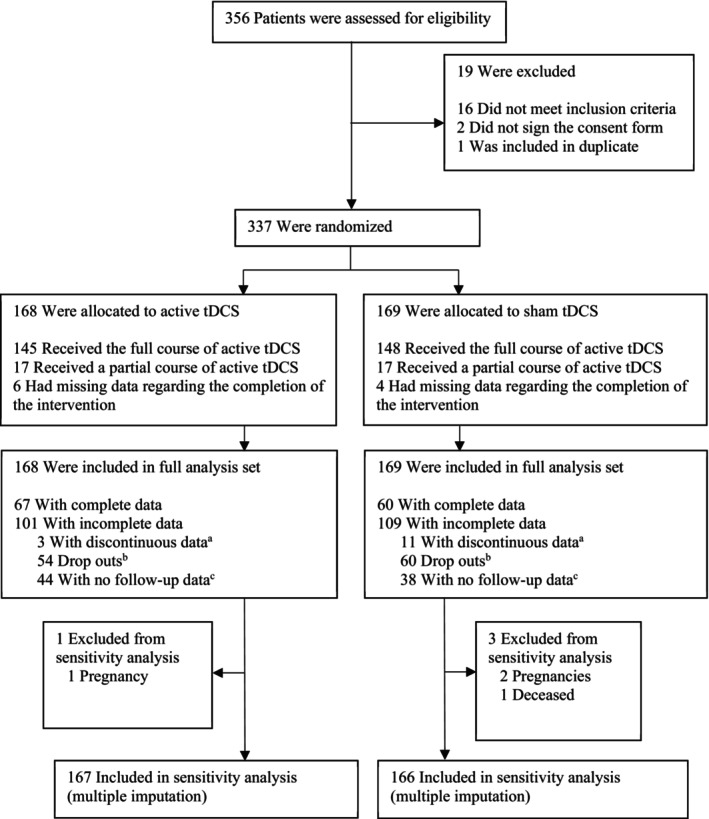
CONSORT diagram showing the flow of participants through each stage of a randomized trial.

From October 2015 to January 2022, of 356 patients initially screened for enrollment, 340 met the inclusion criteria, two were finally excluded for failing to sign the consent form and one duplicate was identified, resulting in a total of 337 randomized patients who received the tDCS (*n* = 168) or sham stimulation (*n* = 169) (Figure 1).

At baseline, the randomized participants were primarily male (60.5%) with an average age of 51.3 ± 11.3 years. The demographic characteristics of the two groups were similar at baseline (Table 1). Among alcohol‐related behaviors, the mean age at first alcohol consumption and length of time of problem drinking were similar. Both groups had an average of two attempts to stop or reduce alcohol consumption before inclusion in the study. In the active group, the TAC was slightly lower (84.9 vs. 92.9 g/day), and there were fewer patients considered as very high RDL according to the WHO classification (42.9% vs. 49.7%). However, the number of HDD was similar between groups (25.0 vs. 26.0 HDD/4 weeks).

**TABLE 1:** Baseline patient characteristics.

Patients randomized	Active tDCS	Sham tDCS
Age (years)	51.6 (11.7)	51.0 (10.9)
Sex (women)	67 (39.9%)	66 (39.1%)
Race (White)	159 (94.6%)	162 (95.9%)
Hand laterality (right‐handed)	151 (89.9%)	152 (89.9%)
Level of education (post‐secondary)	102 (60.7%)	92 (54.4%)
Age of first alcohol consumption	16.8 (6.8)	16.2 (4.7)
Duration of problem drinking (years)	16.0 (12.0)	15.7 (11.4)
Total alcohol consumption (g/day)	84.9 (43.0)	92.9 (45.1)
Total monthly heavy drinking days (days over 4 weeks)	25.0 [18.0–28.0]	26.0 [18.0–28.0]
Number of reduction or abstinence attempts	2.0 [1.0–2.0]	2.0 [1.0–3.0]
OCDS score	19.8 (5.4)	21.0 (5.5)
Drinking risk level (WHO)		
Medium	30 (17.9%)	23 (13.6%)
High	66 (39.3%)	62 (36.7%)
Very high	72 (42.9%)	84 (49.7%)
ADS score	12.7 (5.7)	14.1 (6.2)
Family history of alcohol problems (yes)	113 (67.3%)	124 (74.4%)
Previously treated for AUD (with drugs indicated)	96 (57.1%)	111 (65.7%)
Smoking status		
Smoker	105 (62.5%)	102 (60.4%)
Never smoker	20 (11.9)	17 (10.1)
Former smoker	43 (25.6)	50 (29.6)
Psychiatric history	85 (50.6%)	96 (56.8%)
γ‐Glutamyltransferase (IU/L)	60.5 [32.5–139.0]	79.0 [38.0–188.5]
Mean corpuscular volume	94.6 (4.7)	93.6 (8.3)
Alanine aminotransferase (IU/L)	35.0 [22.5–55.0]	37.0 [23.0–65.0]
Aspartate aminotransferase (IU/L)	29.0 [21.0–49.5]	32.0 [23.0–57.0]
Percentage carbohydrate‐deficient transferrin (%)	1.5 [1.1–2.8]	1.9 [1.2–3.7]

*Note*: Data are presented as means (±SD) or number of participants (%).

Abbreviations: tDCS, transcranial direct current stimulation; OCDS, Obsessive Compulsive Drinking Scale; WHO, World Health Organization; ADS, alcohol dependence scale; AUD, alcohol use disorder.

The Bang blinding index was −0.26 (−0.38 to −0.14) for the active tDCS group and −0.68 (−0.77 to −0.59) for the sham tDCS group. In both arms, the CIs were entirely below zero, indicating that participants guessed their treatment allocation no better than chance, consistent with adequate preservation of blinding. However, the χ^2^ test comparing the proportion of correct guesses between groups was statistically significant (*P* < 0.0001), suggesting differential guessing patterns between arms, with participants in the sham group showing greater uncertainty regarding their allocation.

The overall rate of drop out was 58.2% and similar between groups (Figure 1, Tables S2 and S3). All data was analyzed according to the initial group assignment.

### Primary outcomes

**FIGURE 2:**
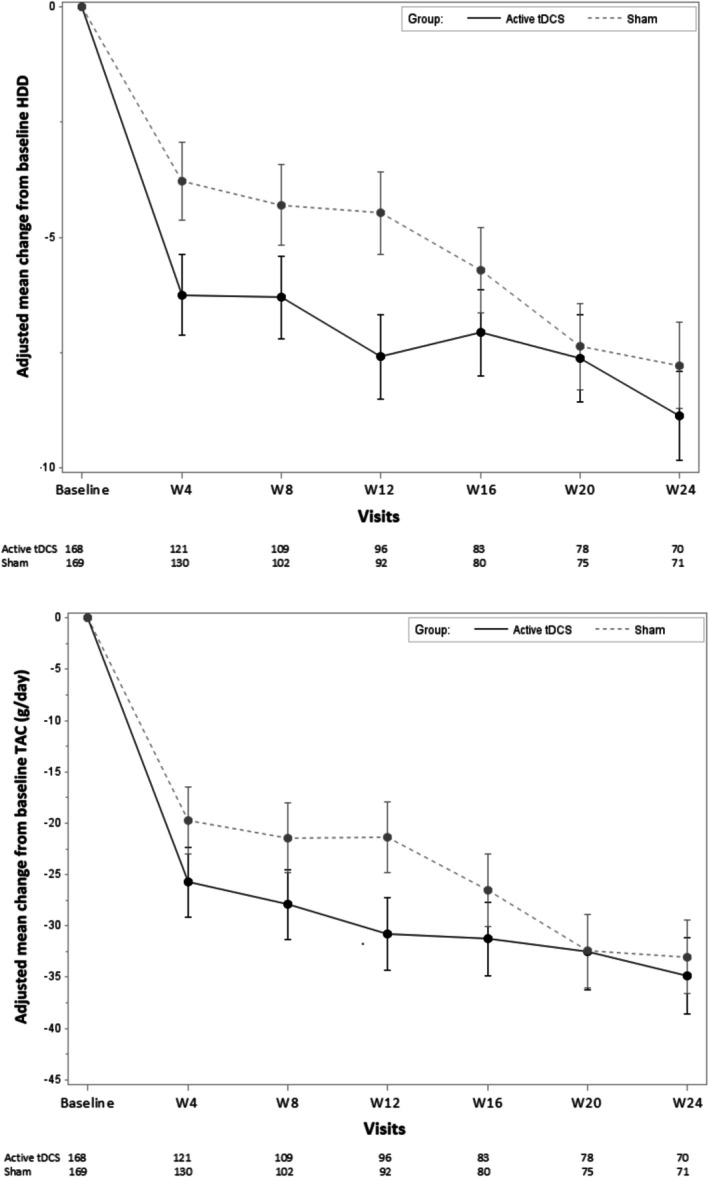
Change in alcohol consumption. (a) Adjusted mean change from baseline in monthly heavy drinking days. (b) Adjusted mean change from baseline in monthly total alcohol consumption (g/day). Baseline data for heavy drinking days and total alcohol consumption were derived from the Timeline Follow Back for the 4‐week period preceding the screening visit.

During the 24‐week follow‐up period, the reduction in the average number of HDD (MMRM) was higher and statistically significant in the active vs. the sham tDCS group (−2.45 HDD/4 weeks; 97.5% CI =−4.86 to −0.05, *P* = 0.022). The reduction in TAC was also higher, but not significant (−5.96 g/day; 97.5% CI = −15.18 to 3.26, *P* = 0.147). The corresponding Cohen's *d* effect sizes for all variables included in the models are reported in Tables 2 and 3. Although the differences in alcohol consumption for both HDD and TAC appear to decrease over time, particularly from week 16 of follow‐up, the interactions between time and treatment were not significant (respectively, *P* = 0.09 and *P* = 0.26) (Figure 2).

**TABLE 2:** Evolution of HDD over the study period in 337 AUD patients using a mixed‐effects model for repeated measures, including site, sex, treatment and time as fixed effects, subjects as random effects, and baseline HDD as a covariate.

	Estimation parameter	Standard error	CI 97.5%	Pr >|t|	Size effect Cohen's *d* CI 97.5%
Treatment (ref = sham tDCS)					
Active tDCS	**−2.45**	**1.07**	**[−4.86 to −0.05]**	**0.022**	−0.35 [−0.80 to 0.09]
Time (ref = W4)					
W8	−0.48	0.66	[−1.97 to 1.01]	0.472	−0.10 [−0.40 to 0.21]
W12	−0.61	0.68	[−2.14 to 0.91]	0.367	−0.13 [−0.44 to 0.19]
W16	−2.02	0.70	[−3.60 to −0.44]	0.004	**−0.41 [−0.74 to −0.09]**
W20	−3.50	0.70	[−5.07 to −1.94]	<0.0001	**−0.72 [−1.04 to −0.40]**
W24	−4.03	0.63	[−5.45 to −2.62]	<0.0001	**−0.83 [−1.11 to −0.54]**
Site (ref = Dijon)					
Nantes	0.42	1.46	[−2.85 to 3.69]	0.771	0.09 [−0.58 to 0.76]
Small centers	−2.50	1.27	[−5.36 to 0.36]	0.050	−0.51 [−1.10 to 0.07]
Tours	−0.62	1.68	[−4.41 to 3.18]	0.716	−0.13 [−0.90 to 0.65]
Sex (ref = male)					
Female	−2.30	1.01	[−4.58 to −0.02]	0.024	**−0.47 [−0.94 to −0.01]**
HDD at baseline	0.81	0.07	[0.64 to 0.97]	<0.0001	**0.17 [0.13 to 0.20]**

*Note*: Interaction time × treatment *P* = 0.09. Bold values indicate statistical significance.

Abbreviations: AUD, alcohol use disorder; HDD, heavy drinking days; Ref, reference; TAC total alcohol consumption; tDCS, transcranial direct current stimulation; W, week.

**TABLE 3:** Evolution of TAC over the study period in 337 AUD patients using a mixed‐effects model for repeated measures, including site, sex, treatment and time as fixed effects, subjects as random effects, and baseline TAC as a covariate.

	Estimation parameter	Standard error	CI 97.5%	Pr >|t|	Size effect Cohen's *d* CI 97.5%
Treatment (ref = sham tDCS)					
Active tDCS	−5.96	4.11	[−15.18 to 3.26]	0.147	−0.33 [−0.83 to 0.18]
Time (ref = W4)					
W8	−1.67	2.48	[−7.24 to 3.90]	0.500	−0.09 [−0.40 to 0.21]
W12	−1.34	2.55	[−7.07 to 4.40]	0.601	−0.07 [−0.39 to 0.24]
W16	−6.88	2.67	[−12.86 to −0.89]	0,010	**−0.38 [−0.71 to −0.05]**
W20	−12.54	2.68	[−18.56 to −6.52]	<0.0001	**−0.69 [−1.02 to −0.36]**
W24	−13.78	2.48	[−19.36 to −8.20]	<0.0001	**−0.76 [−1.06 to −0.45]**
Site (ref = Dijon)					
Nantes	−2.77	5.61	[−15.38 to 9.83]	0.622	−0.15 [−0.84 to 0.54]
Small centers	−6.00	4.92	[−17.04 to 5.04]	0.223	−0.33 [−0.94 to 0.28]
Tours	−13.58	6.52	[−28.23 to 1.07]	0.038	−0.75 [−1.55 to 0.06]
Sex (ref = male)					
Female	−9.30	4.08	[−18.47 to −0.13]	0.023	**−0.51 [−1.02 to −0.01**]
TAC at baseline	0.66	0.05	[0.55 to 0.77]	<0.0001	**0.04 [0.03 to 0.04]**

*Note*: Interaction time × treatment *P* = 0.26. Bold values indicate statistical significance.

Abbreviations: AUD, alcohol use disorder; Ref, reference; TAC total alcohol consumption; tDCS, transcranial direct current stimulation; W, week.

The results of the first sensitivity analysis, which excluded the 10 patients with missing data regarding completion of the intervention, were similar to those of the primary analysis (−2.45 HDD/4 weeks; 97.5% CI = −4.85 to −0.05, *P* = 0.0223, time × treatment interaction *P* = 0.09; −5.96 g/day; 97.5% CI = −15.18 to 3.2, *P* = 0.1471, time × treatment interaction *P* = 0.26). The second sensitivity analysis using multiple imputation yielded comparable results for both HDD (−2.31 HDD/4 weeks; 97.5% CI = −4.61 to −0.003, *P* = 0.0248) and TAC (−6.68 g/day; 97.5% CI = −16.34 to 2.98, *P* = 0.1198). The concordance between the mixed model for repeated measures and the multiple imputation analyses strengthens the robustness of the findings for our primary end points. A third sensitivity analysis was performed using linear regression to compare changes from baseline to week 24. In this analysis, the reduction in HDD was slightly higher, but not statistically significant in the active group compared with the sham group (−0.08 HDD/4 weeks; 97.5% CI = −3.44 to 3.61). Similarly, the reduction in TAC was greater, but not statistically significant, in the active vs. sham tDCS group (−1.92 g/day; 97.5% CI = −14.81 to 10.97).

A sensitivity analysis assuming MNAR applied empirically derived Δ values (HDD: −2 and −2.5 HDD/4 weeks; TAC: −10 g/day and −12 g/day). The estimated treatment effects remained consistent in direction with the primary analyses. For HDD, the treatment effects remained statistically significant [Δ −2: −2.61 HDD/4 weeks (−5.15 to −0.08), *P* = 0.02; Δ −2.5: −2.42 HDD/4 weeks (−4.71 to −0.13), *P* = 0.018], suggesting that the reduction in HDD observed in the primary analysis is reasonably robust to MNAR assumptions. For TAC, applying Δ values of −10 g/day and −12 g/day yielded effect estimates that were similar to the primary analysis and remained non‐significant [Δ −10: −6.37 g/day (−14.79 to 2.04), *P* = 0.09; Δ −12: −6.48 g/day (−14.90 to 1.95), *P* = 0.08], indicating that the null result for TAC was robust to plausible departures from the MAR assumption.

The results of the *post hoc* analyses are presented in the Supporting Information (Tables S4–S6).

### Secondary outcomes

Overall, a two‐level reduction in WHO RDL at 24 weeks (adjusted for sex and baseline WHO RDL) was not significantly different between groups [adjusted OR (aOR) = 0.85 (0.55–1.33)]. Our analysis of a 50%, 70% and 90% reduction in alcohol consumption revealed a numerical effect in favor of the stimulation group for the 70% and 90% categories at 24 weeks (adjusted for sex and baseline TAC) [aOR = 1.22 (0.55–2.72)] and aOR = 1.93 (0.61–6.12), respectively], but the effect was not significant. The changes from baseline for each 4‐week period following the intervention up to week 24 are presented in Table S7. Analyses for abstinence were not possible because of the low number of abstinent individuals. However, when abstinence was collected at the last visit, five individuals (3.0%) in the stimulation group vs. one (1.2%) in the sham group reported abstinence over the previous 4 weeks.

For craving assessments, the results for current craving at 24 weeks were significantly lower in the stimulation group overall [−0.36 (−0.65 to −0.07), *P* = 0.016], but not for maximum craving for past 28 days and for OCDS (Table 4). We found no between‐group differences for the remaining clinical assessments (clinical global impression, mood, cognitive assessment and quality of life).

**TABLE 4:** Secondary outcomes from baseline to 24 weeks: multi‐variate analyses.

Secondary outcome: active tDCS vs. sham tDCS	ITT population		
	Estimation (95% CI)	*P* value	Time × treatment interaction^a^
VAS alcohol instant craving^a^	−0.36 [−0.65 to −0.07]	0.016	0.13
VAS alcohol max craving^a^	0.05 [−0.41 to 0.461]	0.829	0.82
OCDS^a^	−0.09 [−1.122 to 1.03]	0.870	0.21
CGI‐index^b^	0.07 [−0.72 to 0.87]	0.857	0.98
SF‐12 PCS^a^	0.45 [−0.94 to 1.83]	0.526	0.60
SF‐12 MCS^a^	−0.59 [−2.345 to 1.27]	0.531	0.64
HDRS^a^	−0.24 [−1.13 to 0.66]	0.603	0.55
MoCA^a^	−0.056 [−0.39 to 0.27]	0.710	0.93
Aspartate aminotransferase^a^	−2.0 [−9.09 to 5.00]	0.569	0.88
Alanine aminotransferase^a^	−1.9 [−7.28 to 3.43]	0.480	0.71
γ‐Glutamyltransferase^a^	7.82 [−11.17 to 26.82]	0.418	0.50
Mean corpuscular volume^a^	−0.76 [−1.83 to 0.31]	0.165	0.23
Carbohydrate‐deficient transferrin^a^	−0.33 [−0.65 to −0.01]	0.045	0.35
50% reduction OCDS^b^	−0.08 [−1.85 to 1.69]	0.9287	0.24
70% reduction OCDS^b^	−0.06 [−1.87 to 1.76]	0.9525	0.48
90% reduction OCDS^b^	−0.66 [−2.93 to 1.62]	0.5706	0.13
CGI‐S (at least 2 cat.)^b^	0.24 [−0.97 to 1.44]	0.7008	0.23
CGI‐I (at least 2 cat.)^b^	0.01 [−0.85 to 0.87]	0.9740	0.80

Abbreviations: CGI‐I, Clinical Global Impressions‐Improvement; CGI‐S, Clinical Global Impressions‐Severity; HDRS, Hamilton Depression Rating Scale; MoCA, Montreal Cognitive Assessment; OCDS, Obsessive Compulsive Drinking Scale; SF‐12 MCS, 12‐Item Short Form Survey Mental Component Score; SF‐12 PCS, 12‐Item Short Form Survey Physical Component Score; TAC, total alcohol consumption; VAS, visual analog scale.

^a^ Mixed models for repeated measures including site, sex, treatment group as fixed factors, subjects as random factors and each explored variable at baseline as a covariate.

^b^ Generalized linear mixed models with a binomial distribution including fixed effects for sex, baseline TAC (mean daily alcohol consumption over the previous 4 weeks), group, time and the group × time interaction, as well as a subject‐specific random intercept.

For the biochemical alcohol consumption markers, the results for CDT at 24 weeks were significantly lower in the stimulation group [−0.33 (−0.65 to −0.01), *P* = 0.045] (Table 4). The remaining markers did not meet the significance threshold.

### Safety

Over the course of the study period, 69 participants (41.1%) in the active group and 62 participants (36.7%) in the sham group experienced one or more adverse effects. Paresthesia and headache were more frequent in the active group whereas asthenia was more common in the sham group (Table 5). Nine (5.3%) participants in the sham group dropped out of the trial because of adverse events compared to only three (1.8%) in the active group.

**TABLE 5:** Adverse events per group.

AE	Active tDCS (*n* = 168)	Sham tDCS (*n* = 169)
	*n* (%)	*n* (%)
Paresthesia	27 (16.1)	18 (10.7)
Headache	22 (13.1)	14 (8.3)
Asthenia	18 (10.7)	26 (15.4)
Sleep disorders	0 (0.0)	2 (1.2)
Photopsia	2 (1.2)	0 (0.0)
Tinnitus	0 (0.0)	2 (1.2)
Formication	1 (0.6)	1 (0.6)
Burning sensation	1 (0.6)	1 (0.6)
Nausea	1 (0.6)	1 (0.6)
Drowsiness	1 (0.6)	1 (0.6)
Tremor	1 (0.6)	1 (0.6)
Vertigo	1 (0.6)	1 (0.6)
Withdrew from trial because of AE	3 (1.8)	9 (5.3)
Death	0	1 (0.6) by suicide

*Note*: *n* = number of subjects who presented at least one of these AEs. Only AEs having been reported at least twice in one group or for both groups are reported in the table (with the exception of death).

Abbreviations: AE, Adverse events; tDCS, transcranial direct current stimulation.

## DISCUSSION

After a brief 5‐day course of tDCS, this large randomized trial in individuals with AUD showed a reduction in HDD that persisted over 24 weeks of follow‐up. The intervention also led to a reduction in TAC, although this effect did not remain statistically significant at the end of follow‐up. From a clinical and implementation perspective, these findings suggest that a short, non‐invasive neuromodulation intervention may help support reductions in alcohol intake among individuals with AUD.

The standardized effect size (Cohen's *d*) of tDCS efficacy on HDD reduction was −0.35 (−0.80 to 0.09), indicating a moderate, although non‐significant, effect. To contextualize this result, the effect sizes observed for pharmacological treatments approved in France for reducing alcohol consumption are generally small. A recent Cochrane review reported a small, non‐significant effect size for baclofen on HDD (standardized mean difference −0.18; 95% CI = −0.48 to 0.11), and meta‐analytic data for nalmefene similarly indicate a statistically significant small effect size (Hedges' g = −0.20; 95% CI = −0.30 to −0.09) [24, 25]. Although the present trial provides only preliminary evidence for tDCS, our findings suggest that tDCS may hold potential as an intervention of interest for AUD, with an effect size that appears comparable to, or greater than, that reported for currently approved pharmacotherapies.

The magnitude of the reduction observed may appear modest in absolute terms (~2–3 fewer HDD per month). However, even moderate reductions in heavy drinking carry meaningful clinical benefits, including lower risk of alcohol‐related harm, improved functioning and reduced healthcare utilization [20, 26]. It is also important to interpret these effects in the context of the study design, which involved a very brief stimulation period followed by a long follow‐up interval without additional treatment or maintenance sessions. In addition, although our results could seem modest from a clinical perspective, a study performed from a microsimulation model using real‐life observational data predicted that reducing HDD by 20 per year (i.e. 1.5/4 weeks) would be associated with considerable differences in terms of harmful events avoided [27].

Among our exploratory outcomes, the reduction in CDT suggests that the biological markers were consistent with the decrease in alcohol consumption observed in the active tDCS group. This finding is supported by literature indicating that CDT has stronger diagnostic performance than other biomarkers [28]. We also observed a reduction in craving from baseline to the 24‐week follow‐up visit, consistent with previous studies showing the effect of stimulation on craving [17, 29, 30]. However, no reduction was found for the other craving measures, and these results should, therefore, be interpreted with caution.

The REDSTIM study has a number of strengths. First, it is the largest tDCS study ever carried out in AUD or in other addictions. Our results were also obtained with a very brief treatment period during which tDCS proved to be well‐tolerated. Our safety results confirm the excellent safety profile of tDCS, with only 41% of patients reporting adverse effects vs. 68% to 81% of patients in recent drug trials involving medications that are currently approved for alcohol reduction [31, 32, 33]. In terms of our analytic approach, we used up‐to‐date and recommended statistical methods (intention‐to‐treat, MMRM and multiple imputation), and the rigorous sensitivity analysis found that our co‐primary outcomes were consistent [34, 35].

We acknowledge several limitations to our study. The first and most challenging was the high level of dropout and missing data. Nevertheless, these issues are usual among AUD populations and are the most frequent limit in the literature on AUD [35]. The fact that our treatment was delivered over five consecutive days and that there were no additional treatments over the following 24 weeks was likely a factor in the lack of follow‐up for many patients. Furthermore, there was no additional treatment during follow‐up, and we did not implement motivational measures such as behavioral interventions or financial compensation, which are systematically used in pharmacological studies aiming to reduce alcohol consumption. Loss to follow‐up has several implications for the interpretation of our findings, including a reduction in statistical power, a higher likelihood of non‐significant results and a reduced ability to detect true treatment effects. Estimated effects may also be overestimated or underestimated, particularly if missing data are MNAR. The primary analyses relied on the MAR assumption, which was considered the most plausible mechanism in this context. In contrast, the assumption of missing completely at random (MCAR) was deemed unrealistic. Although participants were informed of and had accepted the study procedures, including monitoring of alcohol consumption without an abstinence requirement, MNAR mechanisms cannot be definitively excluded. For example, missed visits could occur during periods of acute intoxication. However, such mechanisms are unlikely to have been systematic, as participants were aware of and had agreed to the study procedures and alcohol monitoring. The observed attrition rate may additionally represent a deviation from a strict intention‐to‐treat approach. Accordingly, the results must be interpreted with caution, and future trials with improved retention strategies are needed to confirm the effects of tDCS before it can be considered for broader implementation in the treatment of AUD. Second, the co‐primary end points were based on self‐reported alcohol consumption. Although self‐report remains the only feasible method for collecting drinking data in outpatient settings, and drinking diaries are widely used for daily monitoring, we cannot ensure that entries were completed contemporaneously. This approach may also be subject to social desirability bias, particularly in the context of the therapeutic relationship. Third, no adjustment for multiple testing was applied to the secondary outcomes, therefore, these findings should be interpreted with caution, as some associations may not remain statistically significant after appropriate correction for multiplicity. Fourth, the representativeness of the study population cannot be ensured, which is a well‐known limitation of controlled clinical trials. As is often observed in AUD research, participants were predominantly male and most were of European ancestry. In addition, the study enrolled individuals who were motivated to reduce their alcohol consumption rather than those aiming for abstinence. Therefore, the findings may not be generalizable to all individuals with AUD, particularly those who differ in demographic characteristics or treatment goals. These analytical limitations, including the possibility of insufficient statistical power and findings that may not remain significant after correction for multiplicity, underscore the need for independent replication of these results.

## CONCLUSION

Following a brief 5‐day course of tDCS, this large‐scale trial in individuals with AUD demonstrated a sustained reduction in HDD up to 24 weeks after treatment. Although these findings require independent replication, they, together with the favorable safety profile observed, support further investigation of this approach for the therapeutic management of AUD, for which effective treatment options remain limited. To enhance therapeutic efficacy, future trials could explore extended stimulation protocols or alternative strategies such as personalized dosing regimens, optimized electrode placement or home‐based interventions. Integrating standardized adherence and motivational support measures, which are best practices in addiction medicine, would also be valuable.

## AUTHOR CONTRIBUTIONS

**Benoit Trojak:** Conceptualization; funding acquisition; investigation; data curation; writing—original draft; writing—review and editing. **Anne Sauvaget:** Investigation; writing—review and editing. **Wissam El Hage:** Investigation; writing—review and editing. **Thomas Wallenhorst:** Investigation; writing—review and editing. **Benjamin Rolland:** Investigation; writing—review and editing. **Philippe Nubukpo:** Investigation; writing—review and editing. **Ghina Harika‐Germaneau:** Investigation; writing—review and editing. **David Szekely:** Investigation; writing—review and editing. **Julie Giustiniani:** Investigation; writing—review and editing. **Marc Auriacombe:** Investigation; writing—review and editing. **Georges Brousse:** Investigation; writing—review and editing. **Sébastien Guillaume:** Investigation; writing—review and editing. **Maxime Bubrovszky:** Investigation; writing—review and editing. **Benjamin Petit:** Conceptualization; investigation; writing—review and editing. **Clémence Cabelguen:** Investigation; writing—review and editing. **Hussein El Ayoubi:** Investigation; writing—review and editing. **Suzanne Rankin:** Writing—original draft; writing—review and editing. **Agnès Soudry‐Faure:** Conceptualization; methodology; formal analysis; data curation; writing—review and editing. **Karine Goueslard:** Conceptualization; methodology; formal analysis; data curation; writing—review and editing. **Anastasia Demina:** Conceptualization; investigation; methodology; formal analysis; data curation; writing—original draft; writing—review and editing.

## DECLARATION OF INTERESTS

None.

## CLINICAL TRIAL REGISTRATION

**Clinical trial registration details** ClinicalTrials.gov Identifier: NCT02505126.

## Supporting information


**Table S1:** Inclusion and exclusion criteria.
**Table S2.** Details regarding complete and incomplete follow‐up data.
**Table S3.** Baseline characteristics of included patients depending on the full completion of follow up data.
**Table S4.** Post hoc analyses: subgroup analyses according to sex and baseline severity (based on DSM‐5 levels).
**Table S5.** Evolution of TAC over the study period using a mixed‐effects model including year of recruitment as a covariate to account for potential temporal effects related to the recruitment period.
**Table S6.** Evolution of HDD over the study period using a mixed‐effects model including year of recruitment as a covariate to account for potential temporal effects related to the recruitment period.
**Table S7.** Proportion of individuals with a two‐level reduction in WHO risk drinking level and a 50%, 70%, and 90% reduction in alcohol consumption at each follow‐up time point.

## Data Availability

B.T. had full access to all the data in the study and takes responsibility for the integrity of the data and the accuracy of the data analysis. The study protocol is provided in the online‐only supplement. Anonymized participant data will be made available at the end of the trial for research purposes, on request to the corresponding author. Proposals will be reviewed and approved by the sponsor and the corresponding author. Once the proposal has been approved, data can be shared via a secure online platform (filesender RENATER) after signing a data transfer agreement. All anonymized data will be available for at least 15 years from the end of the trial.
