# Supplementary material for: Efficacy and safety of transcranial direct current stimulation in alcohol use disorder: A randomized controlled triple‐blind trial
Source: Addiction. 2026 May 6;121(8):2141–53. doi: 10.1111/add.70461 (PMC13357871; doi:10.1111/add.70461)
Supplement: Supplementary file 1 — Table S1: Inclusion and exclusion criteria. Table S2. Details regarding complete and incomplete follow‐up data. Table S3. Baseline characteristics of included patients depending on the full completion of follow up data. Table S4. Post hoc analyses: subgroup analyses according to sex and baseline severity (based on DSM‐5 levels). Table S5. Evolution of TAC over the study period using a mixed‐effects model including year of recruitment as a covariate to account for potential temporal effects related to the recruitment period. Table S6. Evolution of HDD over the study period using a mixed‐effects model including year of recruitment as a covariate to account for potential temporal effects related to the recruitment period. Table S7. Proportion of individuals with a two‐level reduction in WHO risk drinking level and a 50%, 70%, and 90% reduction in alcohol consumption at each follow‐up time point. [file ADD-121-2141-s001.docx]

[**Table S1: Inclusion and exclusion criteria** 2](#_Toc223705824)

[**Table S2: Details regarding complete and incomplete follow-up data** 3](#_Toc223705825)

[**Table S3: Baseline characteristics of included patients depending on the full completion of follow up data** 4](#_Toc223705826)

[**Table S4: Post hoc analyses: subgroup analyses according to sex and baseline severity (based on DSM-5 levels)** 5](#_Toc223705827)

[**Table S5: Evolution of TAC over the study period using a mixed-effects model including year of recruitment as a covariate to account for potential temporal effects related to the recruitment period** 6](#_Toc223705828)

[**Table S6: Evolution of HDD over the study period using a mixed-effects model including year of recruitment as a covariate to account for potential temporal effects related to the recruitment period** 6](#_Toc223705829)

[**Table S7: Proportion of individuals with a two-level reduction in WHO risk drinking level and a 50%, 70%, and 90% reduction in alcohol consumption at each follow-up time point** 7](#_Toc223705830)

# **Table S1: Inclusion and exclusion criteria**

| **Inclusion criteria** | (1) individuals over 18 years of age,  (2) who met criteria for mild to severe AUD based on the Diagnostic and Statistical Manual of Mental Disorders Fifth edition (DSM-5),  (3) seeking to reduce their alcohol consumption,  (4) who had at least one previous attempt of supervised drinking reduction or abstinence maintenance. This last criterion aimed to ensure that participants had attempted the usual methods to treat AUD before consenting to participate in an experimental study. |
| --- | --- |
| **Exclusion criteria** | (1) a breath alcohol concentration > 0 milligrams per liter of exhaled air at the inclusion visit (to ensure informed consent was not influenced by the effects of alcohol)  (2) < 6 heavy drinking days (HDD; a day with alcohol consumption ≥ 60 g for men and ≥ 40 g for women) in the previous 4 weeks;  (3) an average alcohol consumption below the medium risk level according to the WHO in the previous 4 weeks (≤ 40 g/day for men; ≤ 20 g/day for women);  (4) > 3 days of abstinence prior to inclusion;  (5) a revised Clinical Institute Withdrawal Assessment for Alcohol (CIWA) score ≥ 10 (indicating the need for medication-supported detoxification);  (6) any treatment with disulfiram, acamprosate, topiramate, baclofene, naltrexone, or nalmefene over the month prior to inclusion;  (7) any previous episode of pre-delirium tremens or delirium tremens;  (8) DSM-5 criteria for substance use disorder other than alcohol or tobacco;  (9) acute psychiatric disorders that would require hospitalization and/or immediate adjustment of psychotropic medications;  (10) major depression, as defined by the Hamilton Depression Rating Scale (HDRS) greater ≥ 24;  (11) any change in psychotropic medication over the previous month;  (12) severe chronic psychiatric disorders including schizophrenia, paranoia, or bipolar disorder type I and II;  (13) advanced liver, kidney, cardiac, or pulmonary disease or other acute serious or unstable medical conditions that would compromise participation in the study according to the physician’s judgment;  (14) contraindications to tDCS, e.g., metal in the head or medical devices implanted in the brain;  (15) individuals who are pregnant or lactating;  (16) individuals of childbearing potential with a positive urine β– human chorionic gonadotrophin pregnancy test at inclusion;  (17) concurrent participation in another trial, employees of the investigator or trial site, or patients protected by law;  (18) individuals who were not covered by national health insurance;  (19) individuals, who were not able to complete the Alcohol Timeline Followback (TLFB) at inclusion or to complete their daily alcohol consumption in a diary during the 24-week follow up period;  (20) individuals who refused to sign the “safety agreement”. The “safety agreement” was a written contract specifying that if a participant comes to the hospital for a visit or a tDCS session in their own vehicle with a breath alcohol concentration > 0.25 milligrams per liter of exhaled air (which prohibits a person from driving a car in France), the participant agrees to give his/her car keys to the medical staff and authorizes the staff to call a family member/friend to take the patient home if they are unable to use public transport. |

# **Table S2: Details regarding complete and incomplete follow-up data**

|  | tDCS  n = 168 | Sham tDCS  n = 169 | Total  n = 337 |
| --- | --- | --- | --- |
| **Complete data** | 67 | 60 | 127 |
| **Incomplete data** | 101 | 109 | 210 |
| - Discontinuous^a^ | 3 | 11 | 14 |
| - Drop out^b^ | 54 | 60 | 114 |
| - Adverse event | 3 | 3 | 6 |
| - Patient withdrawal | 15 | 17 | 32 |
| - Lost to follow-up | 19 | 23 | 42 |
| - Other | 17 | 17 | 34 |
| - No follow-up data^c^ | 44 | 38 | 82 |

^a^ discontinuous data for the drinking diary (either a missed follow-up visit or missing entries in the drinking diary)

^b^ patient withdrawal during the follow-up for various reasons (patient withdrawal, joint decision by patient and investigator to change therapeutic goal for abstinence, adverse event, inclusion error)

^c^ patients who received treatment but did not participate in follow-up for unknown reasons

# **Table S3: Baseline characteristics of included patients depending on the full completion of follow up data**

|  | **Complete follow-up (n=141)** | | **No follow-up (n=82)** | | **Drop-outs (n=114)** | |
| --- | --- | --- | --- | --- | --- | --- |
|  | n | % | n | % | n | % |
| **Male** | 97 | 68.8 | 36 | 43.9 | 71 | 62.3 |
| **Right-handed** | 127 | 90.1 | 71 | 86.6 | 105 | 92.1 |
| **European ancestry** | 134 | 95.0 | 76 | 92.7 | 111 | 97.4 |
| **In relationship** | 99 | 70.2 | 45 | 54.9 | 69 | 60.5 |
| **Children** | 116 | 82.3 | 57 | 69.5 | 88 | 77.2 |
| **Level of education (post-secondary)** | 74 | 52.5 | 46 | 56.1 | 74 | 64.9 |
| **Tobacco** | 74 | 52.5 | 61 | 74.4 | 72 | 63.2 |
| **Family history of alcohol problems** | 96 | 68.1 | 54 | 65.9 | 87 | 76.3 |
| **Previously treated for AUD** | 85 | 60.3 | 52 | 63.4 | 70 | 61.4 |
| **Drinking Risk Level (WHO)** |  |  |  |  |  |  |
| **Medium** | 23 | 16.3 | 19 | 23.2 | 11 | 9.7 |
| **High** | 61 | 43.3 | 21 | 25.6 | 46 | 40.4 |
| **Very high** | 57 | 40.4 | 42 | 51.2 | 57 | 50.0 |
| **Psychiatric history** | 72 | 51.1 | 50 | 61.0 | 59 | 51.8 |
| **History of substance use** | 31 | 22.0 | 23 | 28.1 | 26 | 22.8 |
| **Epilepsy history** | 4 | 2.8 | 6 | 7.3 | 5 | 4.4 |
| **Medical history** | 116 | 82.3 | 53 | 64.6 | 95 | 83.3 |
| **Age** | 53.6+/-10.9 | | 48.7+/-11.5 | | 50.3+/-11.1 | |
| **Age of first alcohol consumption** | 16.4+/-5.3 | | 17.4+/-6.7 | | 16.0+/-5.8 | |
| **Duration of problem drinking (years)** | 17.2+/-12.5 | | 14.5+/-12.0 | | 15.1+/-13.1 | |
| **TAC (g/day)** | 83.7+/-37.1 | | 91.1+/-57.0 | | 92.3+/-41.3 | |
| **HDD (days/month)** | 21.8+/-6.8 | | 21.7+/-8.3 | | 22.4+/-6.1 | |
| **OCDS Score** | 19.4+/-5.0 | | 22.4+/-6.3 | | 20.1+/-5.1 | |
| **ADS Score** | 12.8+/-5.6 | | 15.0+/-7.1 | | 13.1+/-5.5 | |
| **CIWA Score** | 2.5+/-2.6 | | 3.3+/-2.6 | | 2.9+/-2.7 | |
| **MOCA Score** | 27.0+/-2.5 | | 26.4+/-3.5 | | 27.5+/-2.2 | |
| **CDT (%)** | 2.5+/-2.3 | | 2.7+/-2.9 | | 3.03+/-3.1 | |
| **Alanine Aminotransferase (IU/L)** | 43.9+/631.4 | | 50.2+/-39.0 | | 50.7+/-36.4 | |
| **Aspartate Aminotransferase (IU/L)** | 37.7+/-23.7 | | 48.0+/-45.7 | | 46.5+/-35.6 | |
| **MCV (fL)** | 93.1+/-8.6 | | 94.9+/-4.6 | | 94.8+/-5.1 | |
| **GGT (IU/L)** | 112.7+/6147.5 | | 119.8+/-166.1 | | 181.8+/-235.1 | |

# **Table S4: Post hoc analyses: subgroup analyses according to sex and baseline severity (based on DSM-5 levels)**

| **Subgroup analysis** | **HDD, 97.5%CI, p value** | **TAC, 97.5%CI, p value** |
| --- | --- | --- |
| **Sex**   - Males n = 204 - Females n = 133 | -1.10 [-3.70; 1.51] p=0.34  -4.31 [-8.15; -0.47] p=0.01 | -6.14 [-16.94; 4.66] p=0.20  -5.96 [-18.56; 6.64] p=0.29 |
| **Baseline severity (DSM-5 criteria)**   - Mild AUD n = 14 - Moderate AUD n = 68 - Severe AUD n = 255 | -14.19 [-22.40; -5.99] p=0.0003  0.03 [-4.75; 4.81] p=0.99  -1.97 [-4.50; 0.56] p=0.08 | -19.22 [-37.55; -0.88] p=0.02  -2.68 [-20.33; 14.96] p=0.73  -5.81 [-15.97;4.34] p=0.20 |

**DSM-5:** Diagnostic and Statistical Manual of Mental Disorders Fifth edition, **AUD**: Alcohol Use Disorder

# **Table S5: Evolution of TAC over the study period using a mixed-effects model including year of recruitment as a covariate to account for potential temporal effects related to the recruitment period**

**TAC**

|  |  | **Estimation parameter** | **Stardard error** | **p-value** |
| --- | --- | --- | --- | --- |
| **Treatment** (ref=sham tDCS) |  |  |  |  |
|  | Active tDCS | **-5.43** | **3.68** | **0.14 [-13.70; 2.84]** |
| **Inclusion year** (ref=2015) |  |  |  |  |
|  | 2016 | 14.18 | 30.31 | 0.64 |
|  | 2017 | 18.99 | 29.07 | 0.51 |
|  | 2018 | 13.41 | 28.95 | 0.64 |
|  | 2019 | 26.92 | 28.95 | 0.35 |
|  | 2020 | 29.84 | 29.03 | 0.30 |
|  | 2021 | 27.60 | 29.88 | 0.36 |

# **Table S6: Evolution of HDD over the study period using a mixed-effects model including year of recruitment as a covariate to account for potential temporal effects related to the recruitment period**

**HDD**

|  |  | **Estimation parameter** | **Stardard error** | **p-value** |
| --- | --- | --- | --- | --- |
| **Treatment** (ref=sham tDCS) |  |  |  |  |
|  | Active tDCS | **-2.03** | **0.96** | **0.03 [-4.18; 0.13]** |
| **Inclusion year** (ref=2015) |  |  |  |  |
|  | 2016 | 5.86 | 7.90 | 0.46 |
|  | 2017 | 8.99 | 7.57 | 0.23 |
|  | 2018 | 7.68 | 7.53 | 0.31 |
|  | 2019 | 10.00 | 7.53 | 0.18 |
|  | 2020 | 11.33 | 7.54 | 0.13 |
|  | 2021 | 10.21 | 7.75 | 0.19 |

# **Table S7: Proportion of individuals with a two-level reduction in WHO risk drinking level and a 50%, 70%, and 90% reduction in alcohol consumption at each follow-up time point**

| **Follow-up** | **Outcome** | **p-value** |
| --- | --- | --- |
| **Proportion of individuals with a two-level reduction in WHO risk drinking level^a^** | | |
| Week 4 | OR=1.08 95% CI [0.44; 2.66] | NS |
| Week 8 | OR=0.76 95% CI [0.49; 1.19] | NS |
| Week 12 | OR=0.94 95% CI [0.60; 1.45] | NS |
| Week 16 | OR=0.96 95% CI [0.62; 1.50] | NS |
| Week 20 | OR=0.68 95% CI [0.44; 1.06] | NS |
| Week 24 | OR=0.85 95% CI [0.55; 1.33] | NS |
| **Proportion of individuals with a 50% reduction in alcohol consumption^b^** | | |
| Week 4 | OR=1.01 95% CI [0.54; 1.87] | NS |
| Week 8 | OR=0.98 95% CI [0.52; 1.86] | NS |
| Week 12 | OR=1.21 95% CI [0.63; 2.32] | NS |
| Week 16 | OR=1.00 95% CI [0.51; 1.98] | NS |
| Week 20 | OR=0.58 95% CI [0.30; 1.14] | NS |
| Week 24 | OR=0.73 95% CI [0.37; 1.42] | NS |
| **Proportion of individuals with a 70% reduction in alcohol consumption^b^** | | |
| Week 4 | OR=0.94 95% CI [0.40; 2.23] | NS |
| Week 8 | OR=1.05 95% CI [0.48; 2.30] | NS |
| Week 12 | OR=0.94 95% CI [0.43; 2.07] | NS |
| Week 16 | OR=1.12 95% CI [0.51; 2.46] | NS |
| Week 20 | OR=0.75 95% CI [0.35; 1.58] | NS |
| Week 24 | OR=1.22 95% CI [0.55; 2.72] | NS |
| **Proportion of individuals with a 90% reduction in alcohol consumption^b^** | | |
| Week 4 | OR=0.86 95% CI [0.25; 2.93] | NS |
| Week 8 | OR=1.11 95% CI [0.32; 3.78] | NS |
| Week 12 | OR=3.05 95% CI [0.60; 15.59] | NS |
| Week 16 | OR=1.20 95% CI [0.34; 4.23] | NS |
| Week 20 | OR=2.11 95% CI [0.67; 6.59] | NS |
| Week 24 | OR=1.93 95% CI [0.61; 6.12] | NS |

^a^ logistic regression adjusted by sex, center, and explored variable at baseline

^b^ logistic regression adjusted by sex, and explored variable at baseline
